# The role of artificial intelligence in preoperative planning for Total Hip Arthroplasty: a systematic review

**DOI:** 10.3389/frai.2024.1417729

**Published:** 2024-12-23

**Authors:** Javad Khaje Mozafari, Seyed Ali Moshtaghioon, Seyed Mani Mahdavi, Alireza Ghaznavi, Morteza Behjat, Ali Yeganeh

**Affiliations:** ^1^Trauma and Injury Research Center, Iran University of Medical Sciences, Tehran, Iran; ^2^School of Medicine, Shahroud University of Medical Sciences, Shahroud, Iran; ^3^Department of Orthopedic and Trauma Surgery, Shariati Hospital, Tehran University of Medical Sciences, Tehran, Iran; ^4^Department of Orthopedic Surgery, Rasoul-e-Akram Hospital, Iran University of Medical Sciences, Tehran, Iran; ^5^Department of Orthopedic Hip and Knee Surgery, Rasoul-e-Akram Hospital, Iran University of Medical Sciences, Tehran, Iran

**Keywords:** artificial intelligence, preoperative planning, Total Hip Arthroplasty, orthopedic, systematic review

## Abstract

**Background:**

Total Hip Arthroplasty (THA) is a transformative surgical intervention for hip joint disorders, necessitating meticulous preoperative planning for optimal outcomes. With the emergence of Artificial Intelligence (AI), preoperative planning paradigms have evolved, leveraging AI algorithms for enhanced decision support and imaging analysis. This systematic review aims to comprehensively evaluate the role of AI in THA preoperative planning, synthesizing evidence from studies exploring various AI techniques and their applications.

**Methods:**

A systematic search of PubMed, Scopus, and Web of Science databases was conducted to identify relevant articles. Inclusion criteria encompassed studies focusing on AI in THA preoperative planning, including randomized controlled trials (RCTs), observational studies, and comparative studies.

**Results:**

Six studies from China met the inclusion criteria, collectively analyzing 831 patients. AI-assisted planning demonstrated superior accuracy in estimating prosthesis size and positioning compared to traditional methods. However, limitations such as geographic bias and language constraints were noted.

**Conclusion:**

AI-assisted preoperative planning significantly enhances femoral positioning accuracy, providing superior outcomes compared to traditional methods. This improvement in precision, particularly in the placement of femoral and acetabular components, has been consistently observed across studies, making AI an indispensable tool in improving the overall success of Total Hip Arthroplasty. Despite promising findings, further research is warranted to address limitations and optimize the integration of AI technologies into routine clinical practice.

## Introduction

Total Hip Arthroplasty (THA) stands as one of the very successful and transformative surgical interventions, altogether moving forward the quality of life for people enduring hip joint disorders, such as osteoarthritis, avascular necrosis, or hip fractures. As the prevalence of hip joint disorders proceeds to rise universally, the request for THA is rising, setting an expanding burden on healthcare frameworks. Ideal preoperative arranging is basic for the victory of THA, enveloping different perspectives such as implant choice, component position, and patient-specific contemplations (Deleanu et al., 2016; Torini et al., 2023; Tsiampas et al., 2016).

Traditionally, preoperative planning for THA relied on manual measurements, two-dimensional imaging, and the surgeon’s experience. However, with the coming of Artificial Intelligence (AI), there has been a paradigm shift in how preoperative planning is approached. AI, a branch of computer science centered on making intelligent machines that can perform assignments requiring human insights, have shown great potential in revolutionizing healthcare, including orthopedic surgery (Pesapane et al., 2020; Sandeep Ganesh et al., 2022; Sniecinski and Seghatchian, 2018).

AI applications in healthcare have multiplied in later a long time, with a particular accentuation on moving forward diagnostic accuracy, treatment arranging, and generally understanding care. Within the domain of orthopedics, AI has started to play a pivotal part in preoperative arranging for joint substitution surgeries. The application of AI in THA preoperative arranging includes a range of functionalities, counting picture division, three-dimensional remaking, embed choice, and virtual surgery simulations (Keyser et al., 2020; Sembronio et al., 2019).

One of the primary areas where AI excels is in the analysis of medical imaging. Advanced imaging techniques, such as computed tomography (CT) scans and magnetic resonance imaging (MRI), generate vast amounts of data that can be challenging for surgeons to interpret accurately. AI algorithms, particularly deep learning models, have demonstrated remarkable capabilities in automating the segmentation of anatomical structures, providing precise and efficient three-dimensional reconstructions of the hip joint. This capability is invaluable in visualizing the patient’s unique anatomy, aiding surgeons in making informed decisions regarding implant design and size, placement, and orientation (Ambrus et al., 2023; Nordeck et al., 2015; Oliveira-Santos et al., 2023).

Moreover, AI-driven algorithms can analyze large datasets of historical THA cases, identifying patterns and correlations that may not be immediately apparent to the human eye. These algorithms can contribute to the optimization of implant selection based on factors such as patient demographics, anatomical variations, and postoperative outcomes. As a result, AI has the potential to enhance the personalization of THA procedures, moving away from a one-size-fits-all approach to a more tailored and patient-specific methodology (Oliveira-Santos et al., 2023; Dhopte and Bagde, 2023).

The role of AI in THA preoperative planning extends beyond image analysis. Virtual surgery simulations, powered by AI algorithms, allow surgeons to preview and evaluate different surgical scenarios before entering the operating room. This not only enhances surgical preparedness but also enables the identification and mitigation of potential challenges or complications that may arise during the actual surgery. Such simulations contribute to a more strategic and proactive approach to THA, promoting precision and reducing the likelihood of intraoperative errors (Birkhoff et al., 2021; Cheng et al., 2023; Solanki et al., 2021).

This systematic review aims to comprehensively evaluate the existing body of literature on the role of AI in preoperative planning for Total Hip Arthroplasty. We seek to elucidate the current state of knowledge, identify gaps in research, and provide insights that may guide future developments in this rapidly evolving field. Through a meticulous analysis of the literature, we aim to contribute valuable information to orthopedic surgeons, researchers, and policymakers, ultimately fostering the integration of AI technologies into the standard practice of THA preoperative planning for improved patient outcomes and healthcare delivery.

## Methods

This systematic review will follow the PRISMA (Preferred Reporting Items for Systematic Reviews and Meta-Analyses) guidelines to ensure transparency and credibility in reporting. The protocol for this review has been registered in PROSPERO under the registration number CRD42024504639 (Moher et al., 2015).

### Eligibility criteria

#### Inclusion criteria

Our inclusion criteria will ensure that relevant studies addressing the role of Artificial Intelligence in preoperative planning for Total Hip Arthroplasty (THA) are considered.

- *Study focus*: Studies must focus on the use of AI in preoperative planning for THA. This includes research investigating various AI techniques, algorithms, and applications related to the planning phase of THA.
- *Study types:* We will include randomized controlled trials (RCTs), observational studies (cohort and case–control studies), and comparative studies. The inclusion of different study types will allow for a comprehensive understanding of the current state of evidence.
- *Language*: Given our resources and expertise, articles published in English will be included to ensure accurate interpretation and synthesis of the findings.

#### Exclusion criteria

- *Non-relevance:* Studies not directly related to AI in THA preoperative planning will be excluded.
- *Language:* Non-English publications will be excluded to maintain consistency and accuracy in interpretation.
- *Study design*: Case reports, editorials, and conference abstracts will be excluded as they typically lack the depth and rigor required for this systematic review.

### Information sources

We will conduct a systematic and comprehensive search of electronic databases and manual searches of relevant journals. The databases to be searched include:

- PubMed
- Scopus
- Web of Science

### Search strategy

#### PubMed search strategy

(“Artificial Intelligence”[MeSH Terms] OR “Artificial Intelligence”[Title/Abstract] OR AI[Title/Abstract] OR Machine Learning [MeSH Terms] OR “Machine Learning”[Title/Abstract] OR “Deep Learning”[MeSH Terms] OR “Deep Learning”[Title/Abstract]) AND (“Preoperative Planning”[MeSH Terms] OR “Preoperative Planning”[Title/Abstract]) AND (“Total Hip Arthroplasty”[MeSH Terms] OR “Total Hip Arthroplasty”[Title/Abstract])

#### Scopus search strategy

(TITLE-ABS-KEY(“Artificial Intelligence”) OR TITLE-ABS-KEY(“AI”) OR TITLE-ABS-KEY(“Machine Learning”) OR TITLE-ABS-KEY(“Deep Learning”)) AND (TITLE-ABS-KEY(“Preoperative Planning”) OR TITLE-ABS-KEY(“Total Hip Arthroplasty”))

#### Web of science search strategy

TS = (“Artificial Intelligence” OR “AI” OR “Machine Learning” OR “Deep Learning”) AND TS = (“Preoperative Planning” OR “Total Hip Arthroplasty”).

After removing the duplicate:

### Study selection

Two independent reviewers will conduct an initial screening of titles and abstracts to identify potentially relevant articles based on the inclusion and exclusion criteria. Full-text review of these articles will be performed by the same reviewers, applying the eligibility criteria. Any disagreements between reviewers will be resolved through discussion, and if necessary, consultation with a third reviewer.

### Data extraction

A standardized data extraction form will be developed to capture relevant information from included studies based on the eligibility criteria. The form will include fields for study characteristics, participant characteristics, intervention details, outcomes, and quality indicators.

## Results

### Study selection

**Figure 1:**
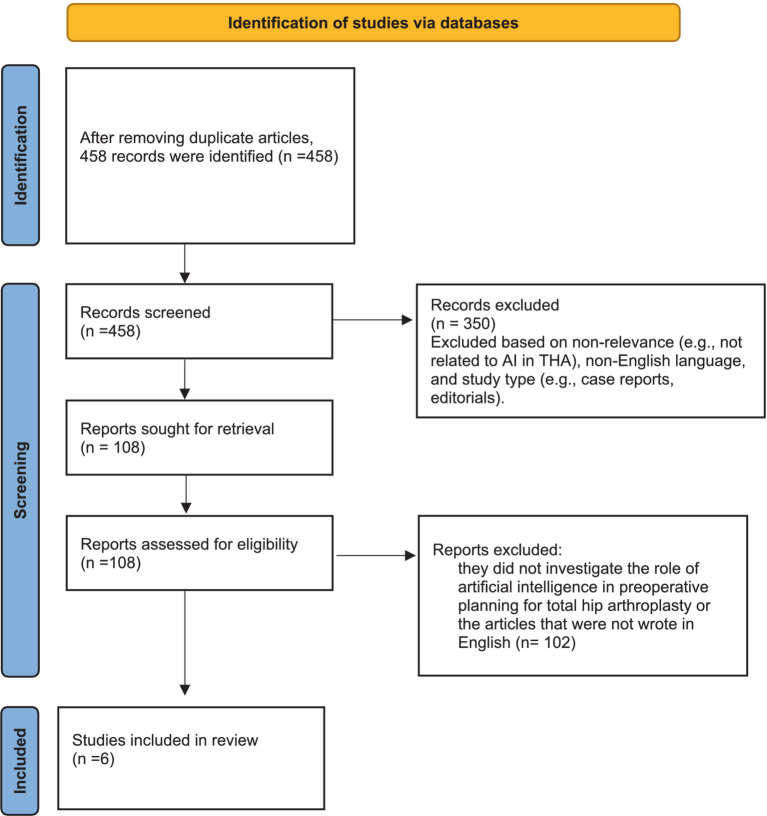
Flowchart of study selection.

The procedure for selecting articles is depicted in Figure 1. In summary, following the elimination of duplicate articles, 458 unique records were obtained from the initial database search for screening titles and abstracts. Among these, 350 were excluded, leaving 108 studies for full-text screening. Among these, 102 were excluded due to their failure to explore the role of artificial intelligence in preoperative planning for total hip arthroplasty or because they were not written in English (Figure 1). Ultimately, 6 studies were deemed suitable for inclusion in the systematic review.

### Demographic data

After search study, there are 6 studies analyzing usage of artificial intelligence on preoperative planning of total hip arthroplasty surgery comparing X-ray template and AI assisted planning by accuracy of femoral stem and acetabular component design, size and position. All of the included studies are performed in China country (Table 1; Chen et al., 2022; Ding et al., 2021; Huo et al., 2021; Wu et al., 2023; Wu et al., 2023; Zhang et al., 2023).

**Table 1:** The extracted data from the sex included studies.

Author, year, country	Study design	Sample size	Duration of Study	Age range	Gender distribution	Intervention details /Group	Control group	Primary outcome	Secondary outcome
Huo et al. (2021), China	RCT	53	10 months	57.4 ± 1.7	male: 57.4%, female: 42.6%	AI HIP group	3D and 2D groups	Compared with the 3D mimics, the accuracy was similar, but the templating time was greatly shortened but Compared with the traditional 2D digital template, its accuracy was much higher, with a slightly longer templating time than that of the digital template	Sex and BMI have no effect on the accuracy of planning acetabular cup and femoral stem by AI HIP but in DDH patients, the accuracy of AI HIP to predict the acetabular cup seemed low
Ding et al. (2021), China	Retrospective study	316	15 months	50.68 ± 12.64	Male: 60.7% female: 39.3%	AI-HIP planning	Manual template planning	Results showed significantly higher consistency of both prosthetic size selection and prosthetic implantation position when AI HIP planning was performed than when traditional manual templating was performed	AI-HIP software achieved an extremely high level of consistency for the femoral stem size, cup size, and femoral osteotomy level. We found significant differences between AI-HIP planning and manual template planning in different BMD scores and Dorr classifications. The prosthetic position methods like level of the rotational center, abduction angle and anteversion of the acetabulum, femoral osteotomy level, depth of the femoral component, femoral offset, and limb length discrepancy all were significantly better calculated in AIHIP group than manual templating method.
Zhang et al. (2023), China	Retrospective cohort	120	15 months	50.53 ± 13.15	Male: 49.6% female: 50.4%	Senior and junior surgeon with AIHIP	Senior and junior surgeon without AIHIP	There was a significant difference in discrepancy in leg length, neck-shaft angle and femoral offset between the healthy side and the affected side, operation duration, decrease in hemoglobin per 24 h, intraoperative radiation exposure frequency and postoperative complications among the patients in junior surgeon group while no differences was seen in senior surgeon	there was no significant difference between senior surgeon and junior surgeon with AIHIP group in terms of the coincidence rate between predicted component size and the actual implantation results on the acetabular cup and femoral stem
Chen et al. (2022), China	RCT	120	17 months	50.68 ± 15.7	Male: 50.8% female: 49.2%	AIHIP preoperative planning	X ray preoperative planning	AIHIP was significantly more accurate than X-ray-based planning in predicting the component size with more high offset stems used	X ray templating tended to underestimate stem size and cup size. Compared with the control group, high offset/varus stems were more commonly used in the AIHIP group. The average time it took to conduct AIHIP planning was significantly more than x-ray planning. Neck length, calcar length, LLD, and offset were measured on radiographs and the difference between observation and control group was not significant. Changes in acetabular offset and global offset were not significantly different between the two groups.
Wu et al. (2023), China	Retrospective study	61	30 months	59.21 ± 10.37	Male: 50.8% female: 49.2%	AI-assisted 3D preoperative planning technology	Traditional two-dimensional X-ray template planning technology	The accuracy of preoperative planning for the acetabular prosthesis and femoral prosthesis in the observation group was significantly higher than in the control group	No statistically significant difference was found in the postoperative abduction and anteversion between the groups. There were significant differences in location of the acetabular prostheses according to Lewinnek and Callanan safe zones, between the two groups. There was a statistically significant difference between the two groups in terms of the postoperative lower-limb length discrepancy, which was significantly improved in both groups compared to preoperative values.
Wu et al. (2023), China	Retrospective study	161	30 months	57.6 ± 10.5	Male: 62.7% female: 37.3%	AI CT scan planning group	Traditional Xray planning group	There were statistically significant differences in the complete accuracy of the acetabular and femoral prostheses and the excellent rate of the femoral prostheses between the two planning methods	There was a statistically significant difference between the two groups in terms of inclination angle and LLD but there was no significant difference in terms of anteversion angle. In the observation group, the percentages of acetabular cups were implanted within the Lewinnek and Callanan safe zones were both values higher significantly higher than control group

Of these 6 studies, 2 studies are randomized controlled trial and 4 studies are retrospective study. Overall number of patients involved in these studies are 831 patients. Age distribution among studies has marked difference which mean age varies from 50.53 (Zhang et al., 2023) to 59.2 years old (Wu et al., 2023) in the studies. The duration of study differed from 10 months (Huo et al., 2021) to 30 months (Wu et al., 2023; Wu et al., 2023) in the studies.

All of the studies divided patients into two groups: traditional 2D X-ray used preoperative planning group and AI HIP assisted group which aims preoperative planning using 3D and CT scan of pelvic. In Zhang et al. study, comparison was done even between senior and junior surgeons to emphasize experience of surgery in preoperative planning of THA (Zhang et al., 2023).

Table 2 summarizes the type of AI systems used, model inputs, and their applications in preoperative planning for Total Hip Arthroplasty across the included studies.

**Table 2:** The type of AI systems used, model inputs, and their applications in preoperative planning for Total Hip Arthroplasty across the included studies.

References	Type of AI	Model inputs	How AI was used
Huo et al. (2021)	AI-HIP software	3D and 2D imaging data (CT scans)	AI-assisted 3D planning for implant accuracy
Ding et al. (2021)	AI-HIP (self-developed)	Pelvic CT scans, BMD scores, Dorr classifications	Planning femoral and acetabular components
Zhang et al. (2023)	AI-HIP	CT scan data	Comparing junior and senior surgeon outcomes using AI-assisted planning
Chen et al. (2022)	AI-HIP system	CT scans, X-rays	Preoperative prosthesis sizing
Wu et al. (2023)	AI 3D-assisted software	3D CT data, Lewinnek and Callanan safe zones	Preoperative planning for acetabular and femoral positioning
Wu et al. (2023)	AI-based CT scan software	CT scan imaging	Preoperative prosthesis placement and accuracy

### Prosthesis size accuracy

The studies declared AIHIP preoperative planning was significantly more accurate than 2D X-ray planning in estimating both acetabular cup and femoral stem component size (all of the studies). Whereas Hou et al. study revealed that in comparison of AIHIP preoperative planning and 3D mimics planning for size of acetabular and femoral components, no significant difference is seen (Huo et al., 2021).

As we can conclude from studies, sex, BMI (Huo et al., 2021), proximal femur morphology (according to Dorr type) and bone mineral density (Ding et al., 2021) are not statistically influencing accuracy of AIHIP for evaluating acetabular and femoral components size. When AIHIP prosthesis size accuracy is compared among DDH and non-DDH patients, no significant difference in predicting femoral stem size is seen while accuracy of AIHIP is statistically lower in predicting acetabular cup size in DDH group compared to non-DDH group (Huo et al., 2021). In one study, the superiority of AIHIP preoperative planning to 2D manual planning was just seen in some selective sizes. Therefore, significant differences were found in only 44, 46, 48, 50, 52, and 54 mm acetabular cups and also 1, 2, 3, 4, 5, 6, and 7 femoral stem sizes whereas in other sizes no meaningful differences were seen (Ding et al., 2021).

In addition, the results came from Chen et al. study shows 2D x-ray planning tends to underestimate acetabular cup size and femoral stem size comparing to AIHIP planning (Chen et al., 2022).

### Prosthesis position accuracy

The position of acetabular and femoral stems was investigated by calculation of parameters like inclination and anteversion of acetabulum and femoral length & offset and comparing them in AIHIP and manual templating groups. The inclination and anteversion of acetabulum were better accounted in AIHIP group than 2D planning group. Also, the level of center of rotation was nearer to its real place in AIHIP planning group. As well as acetabular cup position, femoral stem position was estimated precisely in AIHIP group. The femoral (horizontal) offset, the depth of femoral stem (vertical offset) and femoral osteotomy level were better calculated in AIHIP planning group than manual template planning group. The limb length discrepancy (LLD) was exactly obtained when AIHIP planning group was responsible for preoperative planning (Ding et al., 2021).

Also, Wu et al. study declared the limb length discrepancy in DDH patients was better corrected postoperatively in AIHIP group than X-ray traditional group (Wu et al., 2023).

In another study (Zhang et al., 2023), AIHIP had aimed junior surgeons to have statistically meaningful lower LLD, smaller amount of variation among bilateral femoral offsets and less differences between bilateral neck shaft angles compared to junior surgeons without AIHIP group. While in senior surgeons’ group, there was no significant differences observed in LLD, neck shaft angle and femoral offsets between with AIHIP and without AIHIP groups (Zhang et al., 2023).

Although in Wu et al. (2023) study, AIHIP planning in DDH patients was unable to predict anteversion and abduction angle statistically different from X-ray planning, but the prediction of abduction angle between two groups was significantly different in non-DDH patients. As well the AIHIP accurately located the position of acetabulum based on Callanan and Lewinnek safe zone criteria comparing to traditional planning (Wu et al., 2023).

Unlike other studies, Chen et al. revealed AIHIP was only efficient to calculate femoral offset, while no significant effect was seen estimating acetabular offset, global offset, limb length discrepancy, calcar and neck length comparing to 2D x-ray planning.

### Time of preoperative planning

The templating time of AIHIP group was significantly shorter than 3D mimics planning group whilst it was significantly longer than 2D templating group (Chen et al., 2022; Huo et al., 2021).

### Clinical outcomes

AIHIP was successfully helping junior surgeons’ group to achieve lower duration of THA surgery, lower decrease in Hemoglobin postoperative and lower radiation exposure during surgery, whereas in senior surgery group these effects were not seen. Also, the incidence of aseptic loosening and periprosthetic femoral fracture in junior surgeons with AIHIP group was significantly less than without hip group (Zhang et al., 2023).

In contrast, Chen et al. and Wu et al. studies conclude there is no statistically difference in operation time and blood loss postoperatively between AIHIP group and manual templating group (Chen et al., 2022; Wu et al., 2023; Wu et al., 2023).

## Discussion

Our systematic review confirms that AI-assisted planning using 3D CT scans demonstrates superior outcomes compared to traditional X-ray-based methods. Specifically, AI-based approaches provide significantly better accuracy in determining the size and positioning of femoral and acetabular components. This advantage is primarily due to AI’s ability to leverage three-dimensional reconstructions, which allow for more precise modeling of patient-specific anatomy. These findings were consistent across multiple studies, highlighting the potential of AI in improving surgical precision and outcomes in Total Hip Arthroplasty. In contrast, traditional X-ray-based methods, being two-dimensional, have inherent limitations in accurately representing complex anatomical structures, often leading to less precise planning (Chen et al., 2022; Wu et al., 2023; Wu et al., 2023; Zhang et al., 2023).

Our review encompassed six studies, all conducted in China, analyzing the utilization of AI in THA preoperative planning. These studies predominantly compared traditional two-dimensional (2D) X-ray templating with AI-assisted planning using three-dimensional (3D) reconstructions and computed tomography (CT) scans of the pelvis. Overall, the included studies demonstrated that AI-driven preoperative planning significantly improves the accuracy of estimating both acetabular cup and femoral stem component sizes compared to traditional methods. Notably, AI-based planning exhibited superior performance in certain selective sizes, emphasizing its potential for personalized and precise surgical interventions.

Moreover, AI-assisted planning consistently resulted in more accurate positioning of acetabular and femoral components, as evidenced by parameters such as anteversion, inclination, femoral offset, and limb length. These findings underscore the invaluable role of AI in optimizing implant selection and placement, thereby enhancing surgical outcomes and minimizing postoperative complications.

While AI-driven preoperative planning exhibited clear advantages, particularly for junior surgeons, in terms of shorter templating time, reduced surgical duration, decreased postoperative hemoglobin decrease, and lower radiation exposure, its impact on clinical outcomes varied across studies. Some studies reported significant reductions in complications such as aseptic loosening and periprosthetic fractures, especially among junior surgeons utilizing AI, while others found no statistically significant differences in operation time or blood loss compared to traditional methods.

Overall, our review underscores the transformative potential of AI in revolutionizing preoperative planning for THA, offering enhanced precision, efficiency, and personalized care.

However, further research, particularly in diverse patient populations and surgical settings, is warranted to fully elucidate the long-term clinical benefits and optimize the integration of AI technologies into routine orthopedic practice.

## Strengths and limitation of the study

### Strengths of the study

1. Our study offers a comprehensive assessment of the role of Artificial Intelligence (AI) in preoperative planning for Total Hip Arthroplasty (THA), integrating a systematic review of pertinent literature of included studies.
2. Adherence to the PRISMA (Preferred Reporting Items for Systematic Reviews and Meta-Analyses) guidelines ensures transparency and credibility in reporting, bolstering the methodological robustness of our study.
3. Inclusion of randomized controlled trials (RCTs), observational studies, and comparative studies enables a comprehensive understanding of the current evidence base concerning AI in THA preoperative planning.
4. Utilization of a standardized data extraction form facilitates the capture of relevant information from included studies, thereby streamlining the synthesis of findings and mitigating potential bias.
5. Clear and succinct summaries of key findings, encompassing demographic data, prosthesis size and position accuracy, time of preoperative planning, and clinical outcomes, enhance the accessibility and applicability of our results for clinicians, researchers, and policymakers.

### Limitations of the study

1. All included studies were conducted in China, limiting the generalizability of our findings to other geographic regions with potentially distinct healthcare systems, patient populations, and surgical practices.
2. Our study exclusively included articles published in English, potentially excluding relevant studies published in other languages and introducing language bias.
3. The relatively small number of included studies and patients may impact the statistical power and generalizability of our findings.
4. Variability in study designs, patient populations, and outcome measures across included studies may introduce heterogeneity and complicate direct comparisons. That the variety in methodologies across studies may make it difficult to present a single standardized flowchart
5. Many included studies lacked long-term follow-up data, constraining our ability to assess the durability and sustained impact of AI-driven preoperative planning on clinical outcomes.
6. As the included studies varied in their methodologies, making it challenging to draw direct comparisons on these aspects. Our intention was to provide a broad overview rather than an exhaustive comparison of technical details across studies.

## Conclusion

In conclusion, our systematic review sheds light on the transformative potential of Artificial Intelligence (AI) in preoperative planning for Total Hip Arthroplasty (THA). Through a meticulous examination of the existing literature, we have elucidated the pivotal role of AI-driven approaches in enhancing the precision, efficiency, and personalization of THA procedures. By leveraging advanced imaging technologies and machine learning algorithms, AI facilitates accurate estimation of prosthesis size and optimal positioning of components, thereby improving surgical outcomes and minimizing postoperative complications. While our study reveals promising findings, it also underscores the need for further research to address geographic and language biases, expand sample sizes, and incorporate long-term follow-up data. Nevertheless, the integration of AI technologies into THA preoperative planning represents a significant advancement in orthopedic surgery, promising to revolutionize clinical practice and ultimately benefit patients worldwide.

## Data Availability

The original contributions presented in the study are included in the article/supplementary material, further inquiries can be directed to the corresponding author.
